# Affections of the Nervous System Dependent on Diseases of the Permanent Teeth

**Published:** 1868-10

**Authors:** 


### SELECTED ARTICLES.

### ARTICLE IV.

### *Affections of the Nervous System Dependent on Diseases of the Permanent Teeth.*

Mr. Salter contributes to *Guy's Hospital Reports* a most interesting paper under the foregoing title, which contains much information of direct and practical value to dentists. Disorders of this kind are divided into "those which are reflex, secondary, and remote; and those which are direct, immediate, and from contiguity. In the former category would rank epilepsy, neuralgia, paralysis; in the latter local pain, facial palsy, some forms of amaurosis, &c. In other instances, such as those in which exalted sensibility of the tegumentary nerves of the face, or erratic pains through the maxilary nerves are associated with toothache, it might be difficult to say whether the phenomena are mostly reflex or direct; they probably comprise both conditions. . . . . . The posterior lower molars are but little removed from the tonsils and Eustachian tube, from the parotid region, and from the external auditory passage. The fangs of the upper back teeth are close to the orbit and its all important contents; and more posteriorly they approach the spheno-maxillary fossa and fissure. Thus it is easy to account for the nervous complications which are directly entailed by the spread of inflammation from the periosteum of diseased teeth."

"By far the commonest reflex nervous disturbances to which dental irritation gives rise are neuralgic pains of the head; and this is especially the case where the upper teeth are implicated. In the supra- and infra-orbital nerves, the globe of the eye, the temples, and particularly a spot near the vertex, a little on one side (the side of the affected tooth), —in all these regions 'dental neuralgia' is really very common; and I have observed, not unfrequently, that, where the pain has continued long, the integument has become hot, and tender, and red. . . ."

"The several branches of the trigeminus appear to be the the most susceptible of reflex affection, caused by the dental irritation of one of them; but next to the different elements of the fifth nerve, the branches of the cervical and brachial plexuses are most commonly involved. Thus pains in the neck, shoulder, acromion process, insertion of the deltoid, or bend of the elbow, are by no means uncommon, and with them occasionally a loss of motor power, a weary sense of fatigue in the flexor muscles, and an inability to grasp firmly with the hand. It would really seem that there is, occasionally and in some individuals, a special and exceptional communication between the fifth nerve and those of the arm. Dr. Anstic* has seen two instances in which wounds of branches of the ulnar nerve have caused reflex neuralgia of the fifth nerve. And he remarks upon this circumstance: 'That the mental perception of the patient should, in each of these cases, refer the pain, not to any point in the course of the injured nerve, but to the branches of the trigeminal, affords, in my opinion, a strong suggestion that that portion of the central nervous system with which the trigeminus is directly connected presents some congenital or acquired peculiarity of organization.' This idea is fully borne out by what one occasionally, but only occasionally and exceptionally, sees in the occurrence of brachial neuralgia and paralysis caused by dental irritation of the branches of the fifth nerve.'

"Reflex nervous irritation, dependent upon dental disease, is most uncertain and capricious in its manifestations. One person will suffer much from a comparatively slight cause, while in others the same conditon more severely developed will produce no such result. There is, unquestionably, in some persons a neuralgic diathesis; and it is not improbable also that, in some individual, there may be a congenital or induced peculiarity in the centric or, perhaps, collateral relations of certain nerves, by which the exalted polarity of one may be passed on and so reflected upon another with abnormal facility. In persons obnoxious, to these forms of neuralgia from dental irritation, nothing is so liable to produce an attack as exhaustion or depressed nutrition; and patients will often tell you that the attacks only come on when they are very tired, or have gone long without food."

"*Pain* is only one of the phenomena of reflex dental nerve irritation. It may induce *muscular spasm*, *muscular paralysis*, *paralysis of some of the nerves of special sense*, *perverted nutrition*.

"As regards the teeth themselves which excite this exalted nervous irritability, nearly all their diseases appear capaple of causing this condition. Thus—

* Lettsomian Lectures on Painful Affections of the Fifth Nerve.—*Lancet*. 1866, vol. ii., pp. 31, 32.

"Caries, with or without exposure of the pulp; exostosis; hypertrophy of the crusta petrosa; nodular developments of dentine in the pulp cavity; periostitis, plastic or suppurative; impaction of permanent teeth in the maxillary bones; crowding of teeth from inefficient room—

"Each and all of the above enumerated abnormalities of teeth have caused manifestations of reflex nervous irritation, though, as I have remarked, they may exist in the severest forms without producing any such result."

Mr. Salter then records a series of cases illustrative of the various pathological conditions referred to, of which we are forced to give the following brief abstract:—

### REFLEX AFFECTIONS.

I.—*Facial Neuralgia from Dentine Excrescence in Pulp Cavity.*—A woman, who complained of severe neuralgic pains obviously connected with an upper central incisor. The pain was of a gnawing character, abiding, but not constantly severe: frequently merely a consciousness of the presence of the tooth, but at other times sharp and darting, flashing up the side of the face, &c., through all the branches of the superior maxillary division of the fifth nerve of that side, and considerably augmented by sudden pressure, a tap upon the tooth, or marked change of temperature. The tooth was apparently sound, though somewhat elongated and slightly loose. No exostosis was found on the root after extraction, but, on making a section of it (vertical from side to side), an excrescence of dentine was discovered growing from the side of the pulp cavity and occupying, for a short space, more than half its diameter. The removal of the tooth, though accompanied with a violent paroxysm of neuralgic agony, was followed by total cessation of the pain, which never recurred.

II.—*Cranial Neuralgia from an impacted Canine Tooth.*—Miss B—— had "cut" all the permanent teeth in due course and position, except the left upper canine, the proper space for which was obliterated by the contact of the lateral incisor and first bicuspid. At the time when the right upper canine appeared, a hard swelling was noticed in the palate, on the left side and towards the front of the mouth, and this slowly developed into a prominent rounded ridge, extending obliquely behind the left incisors, and left first bicuspid. No inconvenience was felt up to the age of eighteen, when severe headache, confined to a spot on the vortex towards the left side, attended by local heat, &c., temporarily relieved by pressure, made its appearance. This headache, which was recurrent in its nature, lasted until the patient was twenty-six, no actual pain being felt in the impacted tooth, although the region about it became hot and tender upon the supervention of the headache. The removal of the tooth by Mr. Cartwright—an operation involving much chipping away of the bony cavity in which it was imbedded —was followed by *immediate and permanent relief*, thirty years having elapsed with no return of the symptoms.

III.—*Intense and general Neuralgia from Exostosis on Fangs of Teeth.* Miss B. P——had gone through her first dentition without trouble, but on account of some crowding on the advent of the permanent teeth, an upper and lower bicuspid on either side were extracted to make room. During adolecence she was attacked by neuralgia pains, at first confined to branches of the trigeminus, but afterwards extending to the arms, leg, &c., indeed, nearly the whole body. The teeth, though apparently sound, had a tendency to elongate and spread, especially the upper incisors, with which the pain was at first associated. The offending teeth always gave pain on being slightly struck. Mr. Bell removed, from time to time, the teeth most obvionsly connected with the neuralgia in each instance with temporary relief of the suffering, and in every case the fangs of the extracted teeth were found encrusted with nodular exostosis, though the teeth themselves were free from carries. When Mr. Salter saw Miss P. (1851) only the two lower left bicuspids remained, and these were causing a continuance of the neuralgia, which ceased after their removal. On the fangs of both these teeth were the expected nodules of exostosis. This patient is stated to have been remarkably anæmic, the gums being "like wax stained of the palest pink." and the alveoli remaining white and bloodless for some seconds after extraction before blood enough oozed from the broken vessels to partially fill the hollow sockets.

IV.—*Neuralgia of the arm from Carious Teeth and from undue Pressure of Artificial Teeth.*—In the case of Mrs. E——, caries of any of the lower teeth on the left side has been immediately followed by severe neuralgic pain at a spot, small and circumscribed, on the front of the left forearm, about two inches below the line of flexion. Having now lost all her teeth, and wearing a complete artificial set, whenever the lower denture hurts the jaw on that side the same symptoms are manifested. The right side has never been similarly affected.—*Med. Gazette.*

*To be continued.*

